# The Removal of Arsenic and Its Inorganic Forms from Marine Algae—A Base for Inexpensive and Efficient Fertilizers

**DOI:** 10.3390/molecules29061348

**Published:** 2024-03-18

**Authors:** Jarosław Ostrowski, Alicja Drozd, Rafał Olchowski, Agnieszka Chałabis-Mazurek, Andrzej Sienkiewicz, Agnieszka Kierys, Kinga Morlo, Ryszard Dobrowolski

**Affiliations:** 1 Łukasiewicz Research Network, New Chemical Syntheses Institute, Tysiąclecia Państwa Polskiego Av. 13a, 24-110 Puławy, Poland; jaroslaw.ostrowski@ins.lukasiewicz.gov.pl (J.O.); alicja.drozd@ins.lukasiewicz.gov.pl (A.D.); 2 Department of Pharmacology, Toxicology and Environmental Protection, Faculty of Veterinary Medicine, University of Life Sciences, Akademicka Sq. 12, 20-950 Lublin, Poland; rafal.olchowski@up.lublin.pl (R.O.); agnieszka.mazurek@up.lublin.pl (A.C.-M.); 3 Department of Physical Chemistry, Institute of Chemical Sciences, Faculty of Chemistry, Maria Curie-Sklodowska University, M. C. Sklodowska Sq. 3, 20-031 Lublin, Poland; andrzej.sienkiewicz@mail.umcs.pl (A.S.); agnieszka.kierys@umcs.pl (A.K.); 4 Department of Analytical Chemistry, Institute of Chemical Sciences, Faculty of Chemistry, Maria Curie-Sklodowska University, M. C. Sklodowska Sq. 3, 20-031 Lublin, Poland; kinga.morlo1@gmail.com

**Keywords:** brown seaweed, As, cerium oxide, adsorption, water extraction

## Abstract

Newly synthesized cerium oxide was successfully obtained by the hard templating route. The optimal As(III) and As(V) adsorption onto the studied adsorbent was reached for the initial pH of 4.0 and a contact time of 10 h. The highest static adsorption capacities for As(III) and As(V) were 92 mg g^−1^ and 66 mg g^−1^, respectively. The pseudo-second-order model was well fitted to the As(III) and As(V) experimental kinetics data. The Langmuir model described the As(III) and As(V) adsorption isotherms on synthesized material. The adsorption mechanism of the studied ions onto the synthesized cerium oxide was complex and should be further investigated. The optimal solid–liquid ratio during the proposed aqueous extraction of inorganic As from the *Fucus vesiculosus* algae was 1:50. The optimal dosage of the synthesized cerium oxide (0.06 g L^−1^) was successfully applied for the first time for inorganic As removal from the aqueous algal extract.

## 1. Introduction

Seaweeds (about 10,000 species) are well known for their rich nutritional aspects. They are significant sources of macronutrients with high biological activity (proteins (up to 45% of dry weight), isolated polysaccharides (4–76% of dry weight), dietary fibres, polyunsaturated fatty acids (up to 10% of dry weight), polyphenols (up to 30% of dry weight), sterols, vitamins, carotenoids, etc.), essential micronutrients (e. g. K, Mg, Na, Ca, Fe, Se, Mn, Zn, Cu), and iodine (0.13–1.3 mg g^−^^1^ of dry weight). The composition of seaweeds depends on their type (brown (Phaeophyceae), red (Rhodophyceae), or green (Chlorophyceae), origin, environmental conditions, etc. The high nutrition content in macroalgae is the reason why they and their extracts are used in many areas, such as medicine for iodine deficiency and intestinal disorders, human food (especially in Asia), animal feeds and organic fertilizers. Edible seaweeds, whose global production is ca. 30 mln t each year, possess a relatively low content of heavy metals and As (below 40 mg kg^−^^1^ as total As according to the UE Directive 2002/32/EC). Thus, these algae are used in medicine and food or feed formulas. Currently, scientists are focusing on different methods for incorporating algae into agricultural practices. They have found that adding algae to soil can improve its chemical properties, boost the growth of the microbial community, enhance respiration, and increase photosynthetic activity. Additionally, algae have proven to be effective in bioremediation, helping to clean up pollutants. Various fertilizers made from seaweed are available, such as liquid extracts and seaweed powder for manure. Liquid seaweed extracts have been particularly successful when used as foliar sprays, helping to increase the growth of various crops like herbs, grains, flowers, and vegetables. The use of seaweed fertilizers has significantly grown since they were first introduced in the 1950s due to their ease of use, concentration of nutrients, and longer shelf life compared to seaweed meal. Algae are excellent for fertilizing soil as they release nutrients in a form easily absorbed by plant roots at a steady rate. This means that the nutrient use efficiency of algae can be higher than many chemical fertilizers. Research has also shown that algae can act as an organic slow-release fertilizer, gradually providing plants with essential nutrients like nitrogen and phosphorus. Farmers can expect increased plant growth, better resistance to salt stress, and improved crop yields by using algae as a natural fertiliser. It is a win–win for both the soil and the plants. Unfortunately, macroalgae can easily bioaccumulate toxic substances, such as As, predominantly brown algae. The mechanism of As(V) accumulation by seaweeds is probably based on the similarity of H_2_AsO_4_^-^ with H_2_PO_4_^-^. As(V) can be transported to the algae cells by phosphate transfer channels. In algae, inorganic As species can be partially transformed into the less toxic organic compounds (monomethylarsonic acid (MMA), dimethylarsinic acid (DMA), arsenobetaine (AsB), arsenocholine (AsC), arsenolipids, and arsenosugars). Some factors influence As content in seaweed: the processing and storage conditions, type and age of algae, location, depth of cultivation, or seasonal variability. The highest As content is observed in brown algae. In S. fusiforme, the total As content is 68–149 mg kg^−^^1^ of dry weight, and inorganic As is 42–117 mg kg^−^^1^ of dry weight. Inorganic As species in brown algae can constitute up to 72% of total As species. Using some seaweeds (predominantly brown) in organic fertilizer formulations poses a soil and plant contamination risk by the inorganic As. To solve this problem, methods of inorganic As extraction from seaweeds can be applied before their use [1,2,3,4,5,6,7].

Various liquid media were used for As extraction from macroalgae before determining their species. The most popular are based on methanol, water, diluted aqueous solutions of nitric acid, and aqueous methanol solutions. Typically, extraction processes of As were aided by shaking, ultrasound, or microwaves to increase extraction efficiency. The water turned out to be the best As species extraction solvent from the mentioned media (aqueous ultrasound-assisted extraction (72–93%), aqueous microwave-assisted extraction (49–98%)). This means that seaweeds contain high amounts of polar and easily extractable inorganic As species (As(III) and As(V)). Generally, the inorganic As extraction efficiency depends not only on the extractant type but also on contact time, sample mass/extractant volume ratio, type of algae, and the extraction method. Bioavailable inorganic As species present in macroalgae-based organic fertilizers can easily migrate to the soil and crop plants and enter the food/feed chain, increasing the human/animal exposure risk of toxic As species. This problem can be solved by the aqueous extraction of inorganic As species from macroalgae before their application as an organic fertilizer component with the simultaneous efficient adsorption of As(V)/As(III) from aqueous extracts by the porous solid, such as cerium oxide [3,4,8].

Cerium oxide is an inorganic porous material which is well known for its catalytic (automotive catalysts, ethanol reforming) and sorptive (dextran [9], histidine [10], U [11], phosphates [12], As [13]) properties. They are strictly connected to the surface chemistry of the CeO_2_: the surface oxygen vacancies, Ce(III) and Ce(IV) cations. The Ce(III) cations are essential for the adsorption of As species by the CeO_2_ material. The adsorption mechanism of As onto the CeO_2_ surface depends on the As speciation form. The inorganic As(III) adsorption causes the conversion of Ce(IV) to Ce(III), but the inorganic As(V) adsorption has little impact on Ce(IV) conversion. Additionally, the small CeO_2_ crystallites (which are characterized by a high Ce(III) content on the surface) facilitate faster transfer of the adsorbate to the surface-active sites. The As adsorption capacity of CeO_2_ is also related to its active surface area, which increases and causes the presence of more active sites for As. Unfortunately, it is hard to synthesize this porous solid with the specific surface area exceeding 170 m^2^ g^−^^1^ [13].

In this work, the newly synthesized cerium oxide was used to remove As from a brown algae *Fucus vesiculosus* water extract for the first time. The inorganic As(III) and As(V) adsorption onto the studied material was optimized by the mean of the influence of the aqueous adsorbate solution pH, contact time, and adsorbate concentration. Theoretical pseudo-first-order and pseudo-second-order models were fitted to the adsorption kinetics data. The Langmuir and Frundlich models were used for adsorption isotherms’ description in the studied adsorption systems. Based on the obtained adsorption data, the inorganic As(III) and As(V) adsorption mechanism onto the synthesized cerium oxide surface was proposed. The aqueous extraction procedure of inorganic As from brown algae *Fucus vesiculosus* was proposed and optimized. Finally, the synthesized novel cerium oxide was successfully applied for inorganic As removal from the aqueous algal extract.

## 2. Results and Discussion

### 2.1. Physicochemical Characteristics of Adsorbent

The solid substance selected for the adsorption studies was ceria produced via the hard template method. As it follows from ref. [14], with the conditions given in the Materials and Chemicals section, the method leads to the production of highly porous ceria (the specific surface area, S_BET_ = 187 m^2^ g^−1^, and pore volume, V_p_ = 0.49 cm^3^ g^−1^) in the form of easy-to-handle micro particles built of ceria nanocrystallites (<10 nm), with a surface concentration ratio Ce^3+^:Ce^4+^ equal to 0.26. The ceria selected for this study was a solid of higher porosity to a similar material whose potential for As species adsorption was proven [13]. The comparison of various CeO_2_ materials is presented in Table S1. 

### 2.2. Adsorption Optimization

**Figure 1:**
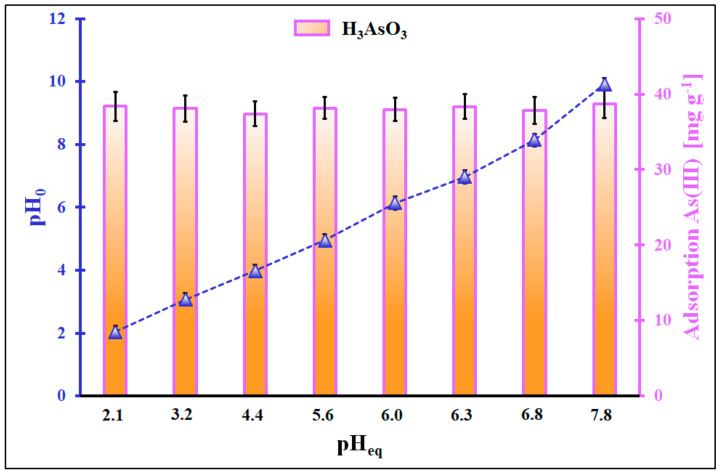
Adsorption of inorganic As(III) species as a function of pH at equilibrium (pH_eq_) and pH of the initial system (pH_0_) as a function of pH_eq_, (m = 5 mg, V = 10 mL, C_0_ = 20 mg L^−1^, pH_0_ range 2.0–9.9, t_eq_ = 24 h, T = (22 ± 2) °C). Error bars denote standard deviations for 4 repeats. The presented As(III) distribution species in the function of solution pH was according to ref. [18].

**Figure 2:**
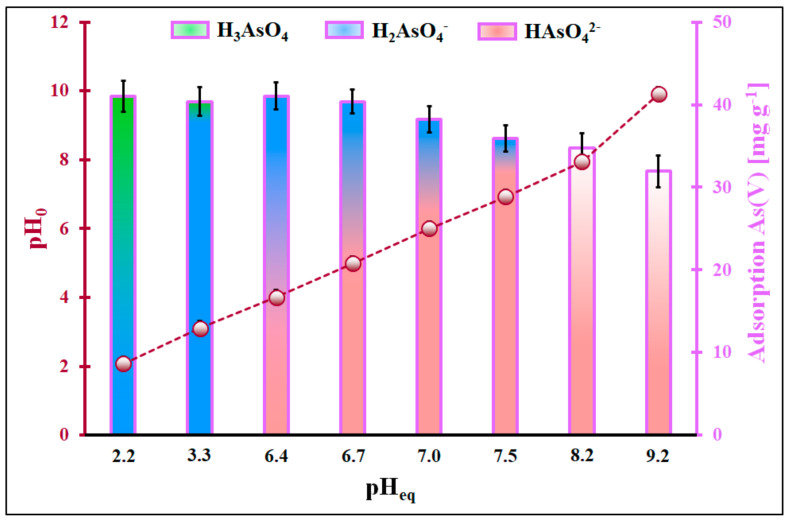
Adsorption of inorganic As(V) species as a function of pH at equilibrium (pH_eq_) and pH of the initial system (pH_0_) as a function of pH_eq_, (m = 5 mg, V = 10 mL, C_0_ = 20 mg L^−1^, pH_0_ range 2.0–9.9, t_eq_ = 24 h, T = (22 ± 2) °C). Error bars denote standard deviations for 4 repeats. The presented As(V) distribution species in the function of solution pH was according to ref. [18].

It has already been shown that the adsorption of inorganic As(III) and As(V) species on the ceria from the aqueous solution highly depends on the adsorbate form and the adsorbent’s charge surface; these, in turn, are highly affected by the pH of the solution [15]. Therefore, the adsorption process was optimized in the initial pH range from 2.0 to 9.9 (Figure 1 and Figure 2). Since the adsorbent was prepared by calcining the XAD7-Ce(NO_3_)_3_-CH_3_OH system in the air at 300 °C, the pH_PZC_ likely equals 8.86 [13]. Hence, in the given conditions, the ceria surface is protonated and positively charged, the inorganic As(III) species exist in a non-ionic form of H_3_AsO_3_, while the inorganic As(V) species can be in two predominant forms in pH < 6.7 in the form of the monovalent anion H_2_AsO_4_^−^ and above pH = 7 in the form of the divalent anion HAsO_4_^2−^ [15,16,17]. The surface of cerium oxide can be considered negatively charged only in a solution with an initial pH = 9.9. 

The pH at the equilibrium of inorganic As(III) species adsorption changes only slightly compared to the initial pH. The adsorption of inorganic As(III) species in the given pH range is very stable, and it reaches 38 mg g^−1^. The mechanism of the adsorption of inorganic As(III) under strong acidic conditions on the ceria surface is somewhat unclear. The ceria surface is positively charged, and the adsorbate is in a non-ionic form (H*_3_*AsO*_3_*); thus, the Coulomb interactions cannot be considered the driving force of the observed phenomenon. An insignificant change in the pH after the adsorption indicates that hydrogen ions probably are not in adsorption competition with the adsorption sites occupied by inorganic As(III) species. The relatively high adsorption could be attributed to the van der Waals interaction between inorganic As(III) species and the organic part of the adsorbent [19] or the presence of Lewis acids in the system, i.e., bicarbonate ions [20]. However, none of the hypotheses mentioned seem to apply to the experiment described here. The adsorption of inorganic As(V) species on ceria in comparison to inorganic As(III) is somewhat different. It is stable from pH_eq_ = 2.2 to pH_eq_ = 5.0 and reads 41 mg g^−1^. When the pH increases, the adsorption diminishes linearly to 32 mg g^−1^ at pH_eq_ = 9.2.

Interestingly, the pH_0_ is almost equal to pH_eq_ for the first two experimental points. However, for pH values higher than 4, the pH_0_ << pH_eq_. This suggests that the hydrogen ions interact with the As(V) species. Above the pH = 4, the HAsO_4_^2−^ can exist next to monovalent ions. The presence of this As form in the system can diminish the activity of H^+^ ions. The high adsorption efficiency of inorganic As(V) species in solutions with pH values smaller than 7 is understandable because inorganic As(V) species are anions that can interact via Coulomb attraction with the positively charged surface of ceria. The drop in the adsorption of inorganic As(V) species is observed in the solution of the initial pH of 6. It can be related to the appearance of divalent inorganic As(V) anions (HAsO_4_^2−^) in the system. A further decrease in the adsorption efficiency of inorganic As(V) species with an increase in the initial pH should be associated with a decrease in the positive charge of the cerium surface. As a result, Coulomb’s repulsions begin to play a large role and the adsorption of inorganic As(V) species drops by 9 mg g^−1^ in the solution of pH_0_~10. On the other hand, it is evident that the overall process of inorganic As(V) adsorption does not rely solely on the electrostatic/Coulomb interaction because even close to the pH_PZC_ of the adsorbent, the adsorption still occurs and can be described as significant. Most likely, in the given system, the inner-sphere complexes are formed between the ceria active centres and oxygen atoms of As(V) species, just like it was reported in ref. [21,22,23,24,25].

**Figure 3:**
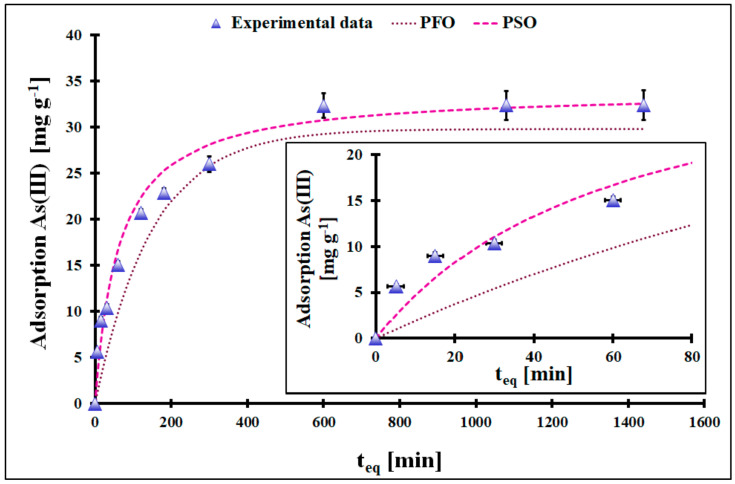
The adsorption kinetic of inorganic As(III) ions onto studied cerium oxide with the linear fitting of PFO and PSO models (m = 5 mg, V = 10 mL, C_0_ = 18 mg L^−1^, pH_0_ = 4, T = (22 ± 2) °C). Error bars denote standard deviations for 4 repeats. Inset represents experimental points for the first 60 min. of solution–adsorbent contact.

**Figure 4:**
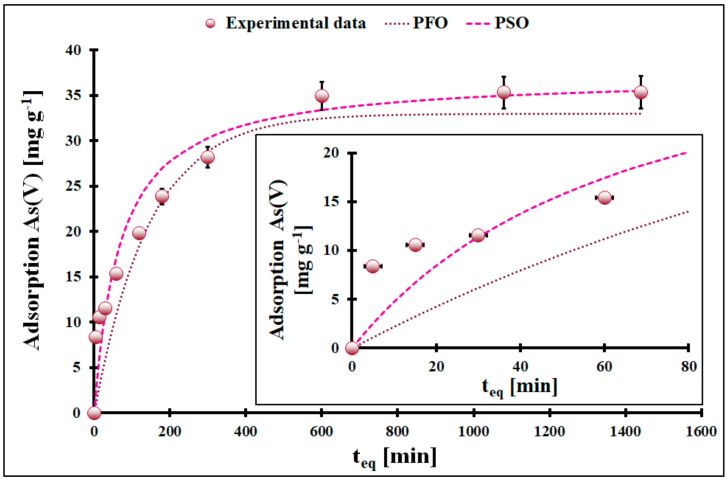
The adsorption kinetic of inorganic As(V) ions onto studied cerium oxide with the linear fitting of PFO and PSO models (m = 5 mg, V = 10 mL, C_0_ = 18 mg L^−1^, pH_0_ = 4, T = (22 ± 2) °C). Error bars denote standard deviations for 4 repeats. Inset represents experimental points for the first 60 min of solution–adsorbent contact.

The course of the adsorption kinetics curves of both inorganic As(III) and As(V) species (Figure 3 and Figure 4) have a similar shape. In the first 200 min, the adsorption rises significantly to reach an adsorption equilibrium at ca. 600 min. So, the adsorption equilibrium is reached after 10 h of adsorbent–solution contact.

The adsorption of inorganic As species on given ceria cannot be considered a fast process. Fitting pseudo-first-order (PFO) and pseudo-second-order (PSO) models into an adsorption kinetics curve is very common in most adsorption kinetics studies, especially in removing contaminants from an aqueous phase. However, both models, like the Freundlich and Langmuir adsorption models, have their limitations and applications. Although the adsorption kinetics mathematically fit very well to the PSO model (R^2^ = 0.9979 for inorganic As(III) and R^2^ = 0.9965 for inorganic As(V)), (Table 1), the conclusion about the adsorption mechanism cannot be expressed without a doubt [26]. In other words, a good fitting of experimental points into a PFO or PSO model is insufficient to discuss the adsorption mechanism. It is worth mentioning that different theoretical calculations indicate that the applicability of both models strongly depends on adsorption system parameters [27,28,29]. It is possible to represent time on a logarithmic scale, which in turn may provide clues regarding the adsorption mechanism of inorganic As(III) and As(V). Then, plots exhibit three distinctive steps. The first stage lasts from 1 min to ca. 50 min. The second one lasts from 50 min to 800 min, and the third stage is from 800 min to 1440 min. The overall adsorption rate will result from well-recognized results in the literature and consecutive steps [14,30,31]. 

The first step is the transport of adsorbate from the bulk solution to adsorbent particles surrounded by the liquid film. Next, the adsorbate molecule diffuses through the film (second step) and into the liquid present in pores (third step, intraparticle diffusion) to finally reach the active sites of the solid, where adsorption and desorption occur (fourth step). The observed experimental adsorption kinetics curves result from these processes co-occurring simultaneously in the system. Since the solution of adsorbate is a complex system, i.e., the inorganic As(III) species solution contains HCl and NaCl, and the inorganic As(V) species solution contains HNO_3_, it is likely that at the solid–solute interface, there is competitive adsorption between chemical individuals in the system. Thus, there is no surprise that the system reaches equilibrium only after 10 h. The simple inorganic anions present in the system, as a result of their preparation method, probably hinder the overall adsorption. Anions such as Cl^-^ or NO_3_^-^ are relatively small compared to inorganic As species; hence, their mobility is much greater, and they can reach the adsorbent surface first and be attracted to the positively charged surface of cerium oxide. Only after a significant period will the competition between adsorbates lead to the significant adsorption of inorganic As species. Additionally, adsorption on cerium oxide in an acidic medium should be considered highly complex since cerium oxide tends to dissolve in highly acidic solutions. Plakhova et al. [32] estimated the solubility of ceria to be at pH = 4.5, equal to 3 × 10^−4^ mol L^−1^ [32].

**Figure 5:**
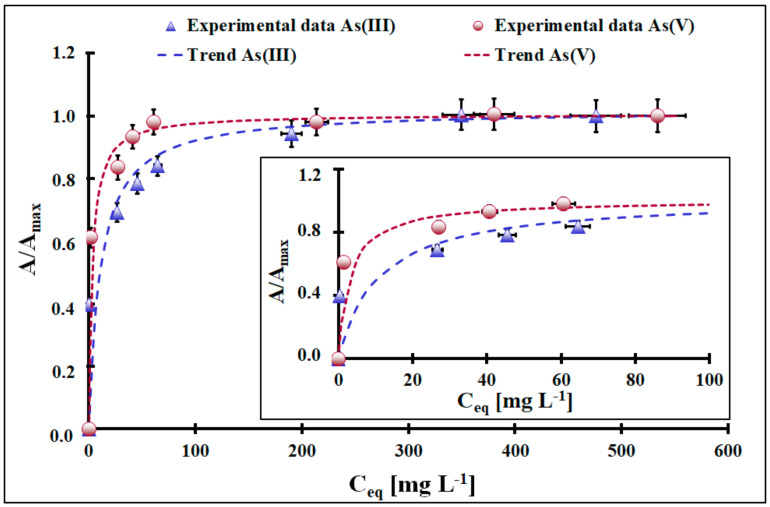
The relative adsorption isotherms for inorganic As(III) and As(V) species from aqueous solution (m = 5 mg, V = 10 mL, C_0_ in a range from 20 mg L^−1^ to 570 mg L^−1^, pH_0_ = 4, t_eq_ = 24 h, T = (22 ± 2) °C, A_max_As(III)_ = 96.1 mg g^−1^, A_max_As(V)_ = 68.5 mg g^−1^, A—adsorption [mg g^−1^], A_max_—adsorption capacity [mg g^−1^]). Error bars denote standard deviations for 4 repeats. Inset represents experimental points for C_eq_ up to 65 mg L^−1^.

The adsorption isotherms of inorganic As species under study at room temperature at pH = 4, measured after 24 h of contact between the adsorbent and the adsorbate, are presented in Figure 5. The acidic conditions were selected deliberately because, at this value, only one form of inorganic As(III) and As(V) is present in the system, i.e., H_3_AsO_3_ and H_2_AsO_4_^-^ [15], respectively. Both the adsorption isotherms presented in Figure 5 show that increasing the adsorbate concentration increases the adsorption up to a certain value of C_eq_, followed by a plateau. The course of isotherms indicates that the adsorbent’s surface is saturated with inorganic As(III) and As(V) species at C_eq_ ca. 300 mg L^−1^ and C_eq_ ca. 90 mg L^−1^, respectively. The maximum adsorption capacity of the adsorption of inorganic As(III) and As(V) species equals 92 mg g^−1^ and 66 mg g^−1^, respectively. These values are high, and the material presented here is within the group of superb inorganic As species adsorbents [13,33,34,35,36,37]. The Langmuir adsorption and Freundlich adsorption models were fitted into experimental points of the adsorption isotherm (Table 2).

Based solely on the coefficient of determination (R^2^), the best fit was obtained for the Langmuir model (R^2^ = 0.9991 for inorganic As(III) and R^2^ = 0.9999 for inorganic As(V)). The superficial analysis of these parameters can conclude that the adsorption of inorganic As species on ceria is a localized process with homogeneous adsorption sites evenly distributed on the adsorbent surface. However, it was already proven [13] that the investigated adsorption system, due to its complexity, goes beyond the assumptions of both models. Thus, the Langmuir and Freundlich adsorption models cannot be used to discuss the adsorption mechanism reliably. On the other hand, based on the experimental results, they can be used to predict adsorption capacity at a given C_eq_.

To sum up, the ceria produced via the hard template method can be used to adsorb inorganic As species from aqueous solutions, including those extracted from macroalgae. Its adsorption capacity is significant, and inorganic As species’ affinity toward ceria has been proven. On the other hand, ceria has its drawbacks, i.e., reaching adsorption equilibrium takes 10 h, and it should be used at a neutral pH. In Table S1, the comparison of As(III) and As(V) adsorption properties between various materials and that studied in this paper, CeO_2_ adsorbent, is presented.

### 2.3. As Aqueous Extraction from Algae

Algae possesses high As content [3]. Part of this metalloid is present in macroalgae as an inorganic form, which is highly toxic for living organisms. The direct application of algae in organic fertilizer formulations could cause the migration of inorganic As compounds from algae into the soil, where they would be deposited and accumulated in crops. Table 3 presents data from the literature and results from our work regarding the total As content and content of different extracted As species in the reference material BCR 279. In general, the extraction of macroalgae with various liquid media (e.g., water) resulted in the removal of inorganic As (mainly As(V) even up to 90% and about 10% As(III)) from this material. As(V) is readily soluble in water, so it is highly mobile in aqueous environments, such as aqueous soil systems. There is a European standard for the determination of inorganic As forms in algae after extraction with 0.07 mol L^−1^ HCl by hydride generation atomic absorption spectrometry (PN-EN 15517) [38]. Only inorganic As can be reduced to volatile hydrides in this technique. Our results suggest that similar amounts of inorganic As are extracted by water and by 0.07 mol L^−1^ HCl. Thus, according to the rules of green chemistry, water is the better liquid medium for the inorganic As removal from seaweeds before their application in agriculture. 

**Figure 6:**
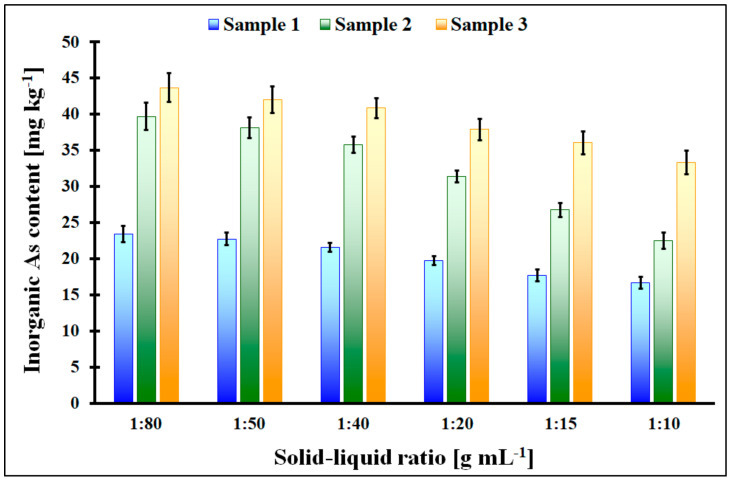
The extractable inorganic As content for the studied algae samples in the function of the solid–liquid ratio; error bars represent standard deviation from 4 repeats.

In Figure 6, the extractable As content for the studied Fucus vesiculosus algae samples in the function of the solid–liquid ratio [g mL^−1^] is shown. For all studied samples, it was observed that the extractable As content was almost stable in the solid–liquid ratio range of 1:80–1:50, and it was lower and lower with further ratio changes from 1:50 to 1:10. So, the optimal solid–liquid ratio, which resulted in the highest inorganic As removal from seaweed and simultaneously was economically justified, was 1:50. Moreover, from our results, it can be concluded that samples 1, 2, and 3 differ from each other in extractable (inorganic) As content. The highest content of inorganic As was observed for the sample denoted as 3 (43 mg kg^−1^), and the lowest was observed for sample 1 (24 mg kg^−1^) for the solid–liquid ratio of 1:50.

Additionally, Table S2 presents the elemental composition of the studied algae samples and BCR 279 before water extraction and the elemental content extracted by water from these algae. The elemental composition of the studied algae differs. The studied algae contained the following macronutrients important in crop growth: Ca (0.3–2.7%), K (1.1–3.5%), Na (1.6–2.4%), Mg (0.7–1.3%), and S (1.4–3.0%). Additionally, the presence of heavy metals such as Al, Cd, Cr, Cu, Fe, Mn, Ni, Zn, and As was detected. According to the presented data, the macronutrients and P were extracted by water to a high extent (>50%). Unfortunately, so were the heavy metals. Their content extracted by water exceeded permissible amounts. The observed phenomenon could be related to the presence of the above-mentioned elements in the form of aqueous soluble chemical species (e.g., Cr, probably as Cr(VI)). On the one hand, the desired macronutrients suggest the reasonable applicability of the studied algae in agriculture, but on the other hand, these toxic elements can be accumulated by crops and pose a real threat to living organisms, including humans.

### 2.4. Inorganic As Removal from the Aqueous Seaweed Extract

**Figure 7:**
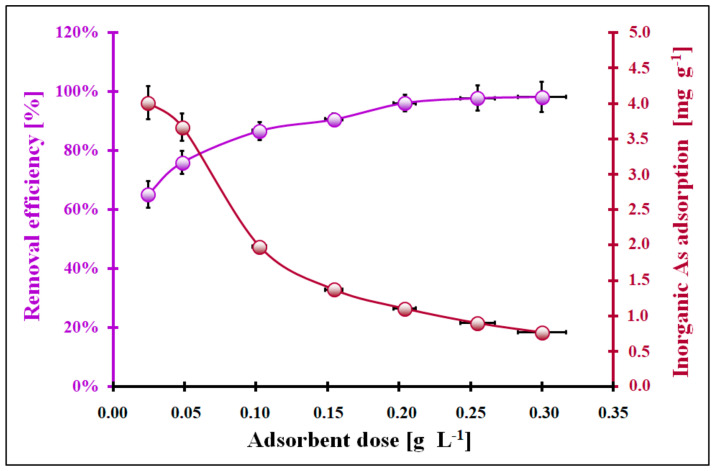
The effect of the cerium oxide dosage on the inorganic As removal efficiency and adsorption capacity (C_0_ = 0.234 mg L^−1^, pH_eq_ = 4, t_eq_ = 24 h, T = (22 ± 2) °C). Error bars denote standard deviations for 4 repeats.

In Figure 7, the inorganic As removal efficiency and adsorption capacity in the function of the CeO_2_ dosage are presented. At higher doses of the adsorbent, the capacity to adsorb inorganic As decreases significantly, going from 4.3 mg g^−1^ to 0.8 mg g^−1^. On the contrary, the inorganic As removal efficiency initially increases from about 64% to 100% within the range of CeO_2_ dosage of (0.02–0.20) g L^−1^, after which, it remains constant. The change in the adsorption capacity of inorganic As with the function of the dosage of CeO_2_ can be related to the aggregation/agglomeration of CeO_2_ particles with its higher dosages [43]. As a result, the collapse in the effective surface area per unit weight (g) of the CeO_2_ could take place and less surface active centres of the adsorbent could be available for the inorganic As. Thus, the adsorption capacity of the studied CeO_2_ towards inorganic As was decreasing. In contrast, the initial increase in inorganic As removal from the aqueous seaweed extract can be related to the increase in the amount of CeO_2_, which provides a larger surface area for inorganic As species to interact with. However, beyond a certain point, the inorganic As removal efficiency is independent of the adsorbent dosage, probably as a result of the maximum possible As adsorption equilibrium shift towards the adsorbent surface. The optimal dosage of CeO_2_ (0.06 g L^−1^) corresponds to the maximum inorganic As removal efficiency, taking into account the highest inorganic As adsorption capacity. From the economic, engineering, and environmental points of view, the usage of the optimal CeO_2_ dosage is reasonable.

## 3. Materials and Methods

### 3.1. Materials and Chemicals

The following chemicals were used during the studies: hydrochloric acid (35–38 wt.%, Chempur, Piekary Śląskie, Poland), nitric acid (65 wt.%, Suprapur, Supelco, Sigma-Aldrich, Saint Louis, MO, USA), sodium hydroxide (>95%, Merck, Darmstadt, Germany), potassium iodide (≥99 wt.%, Sigma-Aldrich, Saint Louis, MO, USA), ascorbic acid (≥98 wt.%, Sigma-Aldrich, Saint Louis, MO, USA), arsenic(III) oxide (>99 wt.%, Sigma-Aldrich, Steinheim, Germany), As(V) SRM solution from NIST (1000 mg L^−1^ (As) as H_3_AsO_4_ in 0.5 mol L^−1^ HNO_3_, Merck, Darmstadt, Germany), and standard Sc solution (1000 mg L^−1^, Supelco, Sigma-Aldrich, Saint Louis, MO, USA). For all experiments, double-deionized water (Merck Millipore, Darmstadt, Germany) was used.

The following materials and samples were used: the standard reference material BCR 279 (*Sea lettuce*) (As_Tot_: 3.02 ± 0.21 mg kg^−1^; JRC, IRMM, Geel, Belgium) and brown algae (*Fucus vesiculosus*) samples with various degrees of contamination, which were obtained from the Gulf of Gdańsk (Poland). In the text, the studied algae samples were denoted as 1, 2, and 3.

### 3.2. Adsorbent Preparation

The adsorption of inorganic As species was performed on highly porous ceria produced via the previously reported hard template method using Amberlite^®^ XAD7 HP (XAD7, Across) polymeric resin [44]. Since the XAD7 microspheres can swell in various solvents [44], this time, XAD7 was soaked with cerium(III) nitrate hexahydrate (Across) dissolved in methanol (POCH) at room temperature. The molality of the ceria precursor solution was 3.46 mol kg^−1^. The amount of ceria precursor solution was adjusted, so there was no liquid outside and beyond saturated XAD7 beads. The XAD7-Ce(NO_3_)_3_-CH_3_OH system was subsequently calcined in air at 300 °C for 360 min. The details about synthesis are given in ref. [14]. 

As it follows from ref. [14], with the conditions mentioned above, this synthesis route led to the production of highly porous ceria (specific surface area, S_BET_ = 187 m^2^ g^−1^, and pore volume, V_p_ = 0.49 cm^3^ g^−1^) in the form of easy-to-handle micro particles built of ceria nanocrystallites (<10 nm), with a surface concentration ratio Ce^3+^:Ce^4+^ equal to 0.26. The ceria produced via the hard template method was selected for this study, because it was already proven that similar ceria have great potential for inorganic As species adsorption [13].

### 3.3. Instrumentation

The As in the aqueous extracts and mineralized samples were determined by a hydride generation ICP-OES spectrometer (Varian 720-ES, Varian, Belrose, Australia). 

The As determination from the aqueous solutions during the adsorption optimization studies on synthesized material was performed by a GF AAS spectrometer (SpectrAA 880Z, Varian, Belrose, Australia). 

The studied algal samples were mineralized in a closed system with microwave assistance from MARS 5 (CEM, Matthews, NC, USA).

### 3.4. Adsorption Experiments

The inorganic As(III) and As(V) species adsorption experiments were conducted at (22 ± 2) °C in static conditions. During the pH influence studies, 5 mg of the studied adsorbent was mixed with 10 mL of an aqueous inorganic As(III) or As(V) species solution with an initial As concentration of 20 mg L^−1^ and an initial pH in the range of 2–10. The resulting suspensions were shaken with a mechanical laboratory shaker at a shaking speed of 148 rpm. After 24 h, the studied adsorbent was separated from the aqueous phase by centrifugation. The initial and final inorganic As(III) or As(V) concentrations in the aqueous phase were determined by the GF AAS technique. The adsorbed inorganic As(III) or As(V) species amount (A [mg g^−1^]) onto the studied adsorbent was calculated as follows:

(1) $$A=\frac{\left(C_{0}-C_{eq}\right)V}{m}$$

where *C_0_* is the initial inorganic As(III) or As(V) species concentration [mg L^−1^], *C_eq_* is the equilibrium inorganic As(III) or As(V) species concentration [mg L^−1^], *V* is the volume of an aqueous inorganic As(III) or As(V) species solution [mL], and *m* is the mass of the used adsorbent [mg].

During inorganic As(III) or As(V) species adsorption kinetics studies, the initial pH of the aqueous inorganic As(III) or As(V) solution (20 mg L^−1^) was 4.0 and adsorption times were in the range of 5–1440 min. The adsorption isotherm onto the studied adsorbent was evaluated for the initial inorganic As(III) or As(V) species concentrations in the range of 20–500 mg L^−1^. 

The kinetic models are expressed by the following equations:

Pseudo first order (PFO):

(2) $$Q_{t}=Q_{eq}\left(1-e^{-k_{1}t}\right)$$

Pseudo second order (PSO):

(3) $$Q_{t}=\frac{Q_{eq}^{2}k_{2}t}{1+{Q_{eq}k}_{2}t}$$

where *Q_t_* is the inorganic As(III) or As(V) adsorption value at time t [mg g^−1^], *Q_eq_* is the inorganic As(III) or As(V) adsorption capacity at an equilibrium state [mg g^−1^], *k_1_* the pseudo-first-order rate constant [g mg^−1^ min^−1^], *k_2_* the pseudo-second-order rate constant [g mg^−1^ min^−1^], and *t* is the adsorption time [min]. 

The isotherm models are expressed by the following equations:

Langmuir model:

(4) $$Q=\frac{C_{eq}k_{L}q_{L}}{{1+C}_{eq}k_{L}}$$

Freundlich model:

(5) $$Q=k_{F}C_{eq}^{n_{F}}$$

where *Q* is the inorganic As(III) or As(V) adsorption capacity at equilibrium state [mg g^−1^], *C_eq_* is equilibrium concentration of inorganic As(III) or As(V) in the solution [mg L^−1^], *k_L_* is the Langmuir constant [L mg^−1^], *q_L_* is the maximum adsorption capacity of inorganic As(III) or As(V) [mg g^−1^], *k_F_* is the Freundlich equilibrium constant [mg^1−nF^ L^nF^ g^−1^], and *n_F_* is the Freundlich exponent.

### 3.5. Inorganic As Extraction Procedure

The standard reference material (BCR 279) and algae samples (1, 2, and 3) were submitted to the extraction procedure by double-deionized water. Various solid–liquid ratios [g mL^−1^] were studied (1:80, 1:50, 1:40, 1:20, 1:15, and 1:10). The proper mass of algae was mixed with 50 mL of water in a glass conical flask. Thus, the obtained suspensions were shaken at 37 °C for 1 h. Next, suspensions were centrifuged (10 min, 7000 rpm), and aqueous phases were filtered by the syringe filter (pore size: 0.45 µm, filter diameter: 30 mm). The total inorganic As was determined in aqueous extracts by the hydride generation ICP-OES technique, and the content of extractable (inorganic) As in the studied algae was calculated by the following equation:

(6) $$Z_{As}=\frac{C_{As}V_{water}}{m_{algae}}\;$$

where *Z_As_* is the extractable (inorganic) As content in algae [mg kg^−1^], *C_As_* is the determined total inorganic As concentration in an aqueous extract [mg L^−1^], *V_water_* is the water volume used for extraction [L], and *m_algae_* is the algae mass used for extraction [kg].

### 3.6. Adsorption of Inorganic As from the Water Algal Extract

Studies of the adsorption capacity of inorganic As (mainly As(V)) on cerium oxide from the water algal extract were carried out. The water extract was filtered, and then, a fixed portion of cerium oxide was added to 5 mL of extract. After reaching an equilibrium, the total inorganic As concentrations were measured using the GF AAS technique. The inorganic As removal efficiencies (*RE* [%]) (Equation (7)) were calculated as follows:

(7) $$RE=\left(\frac{C_{0}-C_{eq}}{C_{0}}\right)\cdot 100\%$$

## 4. Conclusions

The cerium oxide with the specific surface area of 187 m^2^ g^−1^ was successfully synthesized by the hard templating route. It possesses a surface with a Ce^3+^:Ce^4+^ ratio equal to 0.26.

The optimal pH of an aqueous inorganic As(III) and As(V) solution during adsorption onto the synthesized adsorbent was 4.0. The adsorption equilibrium of the studied inorganic As species onto the obtained material was reached after 10 h, and the pseudo-second-order model described it well. The highest static adsorption capacity for inorganic As(III) and As(V) onto the cerium oxide was 92 mg g^−^^1^ and 66 mg g^−^^1^, respectively. The evaluated adsorption isotherms in the studied adsorption systems were well fitted to the Langmuir model. The adsorption mechanism of inorganic As(III) and As(V) ions onto the synthesized cerium oxide was complex due to the nature of adsorbate and adsorbent.

The efficient aqueous extraction of inorganic As from *Fucus vesiculosus* algae before its application in agriculture was proposed for a solid–liquid ratio of 1:50. This extraction procedure agreed with the green chemistry rules. The inorganic As was successfully removed from the water algal extract by 0.06 g L^−^^1^ of the synthesized cerium oxide. This small adsorbent dosage was reasonable from the economic, environmental, and engineering points of view.

## Figures and Tables

**Table 1:** Experimental kinetics fitting results to the pseudo-first-order (PFO) and pseudo-second-order models (PSO). Q_t_ is the inorganic As(III) or As(V) adsorption value at time t [mg g^−1^], Q_eq_ is the inorganic As(III) or As(V) adsorption capacity at an equilibrium state [mg g^−1^], k_1_ the pseudo-first-order rate constant [g mg^−1^ min^−1^], k_2_ the pseudo-second-order rate constant [g mg^−1^ min^−1^], and t is the adsorption time [min].

	Kinetic Model	PFO		
Inorganic As Species		k_1_ [g mg^−1^ min^−1^]	Q_eq_ [mg g^−1^]	R^2^
As(III)		0.0067 *^$^	29.8 * ± 1.1 ^#^	0.9113
As(V)		0.0069 *^$^	33.0 * ± 1.4 ^#^	0.9902
	**Kinetic** **Model**	**PSO**		
**Inorganic** **As Species**		**k_2_ [g mg^−1^ min^−1^]**	**Q_eq_ [mg g^−1^]**	**R^2^**
As(III)		0.00047 *^$^	34.0 * ± 1.5 ^#^	0.9979
As(V)		0.00040 *^$^	37.2 * ± 1.7 ^#^	0.9965

*—mean value from 4 repeats, ^#^—standard deviation from 4 repeats, ^$^—standard deviation close to 0.

**Table 2:** Experimental adsorption isotherm fitting results to the Langmuir and Freundlich models. k_L_ is the Langmuir constant [L mg^−1^], q_L_ is the maximum adsorption capacity of inorganic As(III) or As(V) [mg g^−1^], k_F_ is the Freundlich equilibrium constant [mg^1−nF^ L^nF^ g^−1^], and n_F_ is the Freundlich exponent.

	Isotherm Model	Langmuir		
Inorganic As Species		k_L_ [L mg^−1^]	q_L_ [mg g^−1^]	R^2^
As(III)		0.094 *^$^	96.1 * ± 3.8 ^#^	0.9991
As(V)		0.324 * ± 0.012 ^#^	68.5 * ± 2.7 ^#^	0.9999
	**Isotherm** **Model**	**Freundlich**		
**Inorganic** **As Species**		**n_F_ [a. u.]**	**k_F_ [mg^1-nF^ L^nF^ g^−1^]**	**R^2^**
As(III)		0.13 *^$^	42.9 * ± 1.9 ^#^	0.9898
As(V)		0.083 *^$^	43.1 * ± 1.5 ^#^	0.8732

*—mean value from 4 repeats, ^#^—standard deviation from 4 repeats, ^$^—standard deviation close to 0.

**Table 3:** Reported content of different As species and total arsenic in BCR 279.

Literature	Content of Arsenic Species [mg kg^−1^]			Content of Total Arsenic [mg kg^−1^]	Type of Extract
	DMA	MMA	As_inorg._		
[39]	0.20	0.23	0.74	2.54	methanol + ammonium carbonate
[40]	0.08	0.07	0.52	0.93	methanol:water 1:1
	0.04	0.03	1.20	1.51	nitric acid 2%
[8]	0.08	0.11	0.71	1.12	nitric acid 1%
[41]	0.06	0.23	0.04	0.87	aqueous
[42]	—	—	0.70	1.30	aqueous
Our work	—	—	0.75 * ± 0.03 ^#^	3.10 * ± 0.12 ^#^	aqueous
			0.891 * ± 0.037 ^#^		0.07 mol L^−1^ HCl

*—mean value from 4 repeats, ^#^—standard deviation from 4 repeats.

## Data Availability

The raw/processed data required to reproduce these findings cannot be shared at this time due to technical or time limitations.

## Supplementary Materials

The following supporting information can be downloaded at: https://www.mdpi.com/article/10.3390/molecules29061348/s1, Table S1: Comparison of studied material with literature data regarding specific surface area and As(III)/As(V) adsorption performance. Table S2: Elemental composition of studied algae and BCR-279 before water extraction and elemental content extracted by water from these samples (*—mean value from 4 repeats, #—standard deviation from 4 repeats, LOD [mg kg-1]: Cd (0.02), Pb (2.0), Se (4.0)). References [45,46,47,48,49] are cited in Supplementary Materials.

## References

[1] Cherry P., O’Hara C., Magee P.J., McSorley E.M., Allsopp P.J. (2019). Risks and benefits of consuming edible seaweeds. Nutr. Rev..

[2] Illera-Vives M., Seoane Labandeira S., Iglesias Loureiro L., López-Mosquera M.E. (2017). Agronomic assessment of a compost consisting of seaweed and fish waste as an organic fertilizer for organic potato crops. J. Appl. Phycol..

[3] Reis V.A.T., Duarte A.C. (2018). Analytical methodologies for arsenic speciation in macroalgae: A critical review. Trends Anal. Chem..

[4] Banach J.L., Hoek-van den Hil E.F., van der Fels-Klerx H.J. (2020). Food safety hazards in the European seaweed chain. Compr. Rev. Food Sci. Food Saf..

[5] Camurati J.R., Salomone V.N. (2020). Arsenic in edible macroalgae: An integrated approach. J. Toxicol. Environ. Health B.

[6] Górka B., Korzeniowska K., Lipok J., Wieczorek P.P., Chojnacka K., Wieczorek P., Schroeder G., Michalak I. (2018). The Biomass of Algae and Algal Extracts in Agricultural Production. Algae Biomass: Characteristics and Applications.

[7] Kholssi R., Lougraimzi H., Grina F., Lorentz J.F., Silva I., Castaño-Sánchez O., Marks E.A. (2022). Green agriculture: A review of the application of micro-and macroalgae and their impact on crop production on soil quality. J. Soil. Sci. Plant. Nutr..

[8] Camurati J.R., Londonio A., Smichowski P., Salomone V.N. (2021). On-line speciation analysis of arsenic compounds in commercial edible seaweed by HPLC–UV-thermo-oxidation-HG-AFS. Food Chem..

[9] Ju X., Šmíd B., Johánek V., Khalakhan I., Yakovlev Y., Matolínová I., Matolín V. (2021). Investigation of dextran adsorption on polycrystalline cerium oxide surfaces. Appl. Surf. Sci..

[10] Bercha S., Mali G., Khalakhan I., Skála T., Prince K.C., Matolín V., Tsud N. (2016). Histidine adsorption on nanostructured cerium oxide. J. Electr. Spectr. Rel. Phenom..

[11] Kashyap K., Khan F., Verma D.K., Agrawal S. (2023). Effective removal of uranium from aqueous solution by using cerium oxide nanoparticles derived from citrus limon peel extract. J. Radioanal. Nucl. Chem..

[12] Guo H., Li W., Wang H., Zhang J., Liu Y., Zhou Y. (2011). A study of phosphate adsorption by different temperature treated hydrous cerium oxides. Rare Met..

[13] Lupa J., Morlo K., Dobrowolski R., Legutko P., Sienkiewicz A., Kierys A. (2023). Highly porous cerium oxide prepared via a one-step hard template method as an extremely effective adsorbent for arsenic species removal from water. Chem. Eng. J..

[14] Sienkiewicz A., Chrzanowska A., Kierys A. (2024). Highly Porous Ceria as an Adsorbent for Removing Artificial Dyes from Water. Environ. Process..

[15] Mohan D., Pittman C.U. (2007). Arsenic removal from water/wastewater using adsorbents—A critical review. J. Hazard. Mater..

[16] Martell A.E., Smith R.M. (1982). Protonation Values for Other Ligands. Critical Stability Constants: First Supplement.

[17] Reed J.J. (2020). Digitizing “The NBS Tables of Chemical Thermodynamic Properties: Selected Values for Inorganic and C1 and C2 Organic Substances in SI Units”. J. Res. Natl. Inst. Stand. Technol..

[18] Salameh Y., Al-Lagtah N., Ahmad M.N.M., Allen S.J., Walker G.M. (2010). Kinetic and thermodynamic investigations on arsenic adsorption onto dolomitic sorbents. Chem. Eng. J..

[19] Lee S., Lalhmunsiama L., Thanhmingliana T., Diwakar T. (2015). Porous hybrid materials in the remediation of water contaminated with As(III) and As(V). Chem. Eng. J..

[20] Weerasundara L., Ok Y.-S., Bundschuh J. (2021). Selective removal of arsenic in water: A critical review. Environ. Pollut..

[21] Xu Y.-H., Nakajima T., Ohki A. (2002). Adsorption and removal of arsenic(V) from drinking water by aluminum-loaded Shirasu-zeolite. J. Hazard. Mater..

[22] Zheng Y.M., Zou S.W., Nanayakkara K.N., Matsuura T., Chen J.P. (2011). Adsorptive removal of arsenic from aqueous solution by a PVDF/zirconia blend flat sheet membrane. J. Membr. Sci..

[23] Dudek S., Kołodyńska D. (2022). Arsenate removal on the ion exchanger modified with cerium(III) ions. Physicochem. Probl. Miner. Process..

[24] Tchieda V.K., D’Amato E., Chiavola A., Parisi M., Chianese A., Amamra M., Kanaev A. (2016). Removal of Arsenic by Alumina: Effects of Material Size, Additives, and Water Contaminants. CLEAN—Soil Air Water.

[25] Li R., Li Q., Gao S., Shan J.K. (2012). Exceptional arsenic adsorption performance of hydrous cerium oxide nanoparticles: Part A. Adsorption capacity and mechanism. Chem. Eng. J..

[26] Plazinski W., Dziuba J., Rudzinski W. (2013). Modeling of sorption kinetics: The pseudo-second order equation and the sorbate intraparticle diffusivity. Adsorption.

[27] Plazinski W., Rudzinski W., Plazinska A. (2009). Theoretical models of sorption kinetics including a surface re-action mechanism: A review. Adv. Colloid Interface Sci..

[28] Zhang J. (2019). Physical insights into kinetic models of adsorption. Sep. Purif. Technol..

[29] Azizian S. (2004). Kinetic models of sorption: A theoretical analysis. J. Colloid Interface Sci..

[30] Sahoo T.R., Prelot B. (2020). Chapter 7—Adsorption processes for the removal of contaminants from wastewater: The perspective role of nanomaterials and nanotechnology. Nanomaterials for the Detection and Removal of Wastewater Pollutants.

[31] Tan K.L., Hameed B.H. (2017). Insight into the adsorption kinetics models for the removal of contaminants from aqueous solutions. J. Taiwan Inst. Chem. E.

[32] Plakhova T.V., Romanchuk A.Y., Yakunin S.N., Dumas T., Demir S., Wang S., Minasian S.G., Shuh D.K., Tyliszczak T., Shiryaev A.A. (2016). Solubility of Nanocrystalline Cerium Dioxide: Experimental Data and Thermodynamic Modeling. J. Phys. Chem. C.

[33] Sakthivel T.S., Das S., Pratt C.J., Seal S. (2017). One-pot synthesis of a ceria–graphene oxide composite for the efficient removal of arsenic species. Nanoscale.

[34] Lunge S., Singh S., Sinha A. (2014). Magnetic iron oxide (Fe_3_O_4_) nanoparticles from tea waste for arsenic removal. J. Magn. Magn. Mater..

[35] Cui H., Li Q., Gao S., Shang J.K. (2012). Strong adsorption of arsenic species by amorphous zirconium oxide nanoparticles. J. Ind. Eng. Chem..

[36] Xu W., Wang J., Wang L., Sheng G., Liu J., Yu H., Huang X.J. (2013). Enhanced arsenic removal from water by hierarchically porous CeO_2_–ZrO_2_ nanospheres: Role of surface- and structure-dependent properties. J. Hazard. Mater..

[37] Han C., Pu H., Li H., Deng L., Huang S., He S., Luo Y. (2013). The optimization of As(V) removal over mesoporous alumina by using response surface methodology and adsorption mechanism. J. Hazard. Mater..

[38] (2008). Foodstuffs—Determination of Trace Elements—Determination of Inorganic Arsenic in Seaweed by Hydride Generation Atomic Absorption Spectrometry (HG AAS) after Acid Digestion.

[39] Hsieh Y.J., Jiang S.J. (2012). Application of HPLC-ICP-MS and HPLC-ESI-MS procedures for arsenic speciation in seaweed. J. Agric. Food Chem..

[40] Foster S., Maher W., Krikowa F., Apte S. (2007). A microwave-assisted sequential extraction of water and dilute acid soluble arsenic species from marine plant and animal tissues. Talanta.

[41] Pell A., Márquez A., López-Sánchez J.F., Rubio R., Barbero M., Stegen S., Queirolo F., Díaz-Palma P. (2013). Occurrence of arsenic species in algae and freshwater plants of an extreme arid region in northern Chile, the Loa River basin. Chemosphere.

[42] Caumette G., Koch I., Estrada E., Reimer K.J. (2011). Arsenic speciation in plankton organisms from contaminated lakes: Transformations at the base of the freshwater food chain. Environ. Sci. Technol..

[43] Mashkoor F., Nasar A., Inamuddin Asiri A.M. (2018). Exploring the reusability of synthetically contaminated wastewater containing crystal violet dye using tectona grandis sawdust as a very low-cost adsorbent. Sci. Rep..

[44] Sienkiewicz A., Kierys A. (2021). Polymer templated production of highly porous cerium oxide in direct temperature driven transformation of cerium(III) salt. Micropor. Mesopor. Mat..

[45] Hu W., Yang L., Shao P., Shi H., Chang Z., Fang D., Wei Y., Feng Y., Huang Y., Yu K. (2022). Proton Self-Enhanced Hydroxyl-Enriched Cerium Oxide for Effective Arsenic Extraction from Strongly Acidic Wastewater. Environ. Sci. Technol..

[46] Pang J.H., Liu Y., Li J., Yang X.J. (2019). Solvothermal synthesis of nano-CeO_2_ aggregates and its application as a high-efficient arsenic adsorbent. Rare Met..

[47] Bi X., Zeng C., Westerhoff P. (2020). Adsorption of Arsenic Ions Transforms Surface Reactivity of Engineered Cerium Oxide Nanoparticles. Environ. Sci. Technol..

[48] Shao P., Ding L., Luo J., Luo Y., You D., Zhang Q., Luo X. (2019). Lattice-Defect-Enhanced Adsorption of Arsenic on Zirconia Nanospheres: A Combined Experimental and Theoretical Study. ACS Appl. Mater. Interfaces.

[49] Luo J., Luo X., Hu C., Crittenden J.C., Qu J. (2016). Zirconia (ZrO_2_) Embedded in Carbon Nanowires via Electrospinning for Efficient Arsenic Removal from Water Combined with DFT Studies. ACS Appl. Mater. Interfaces.

