# Supplementary material for: The Removal of Arsenic and Its Inorganic Forms from Marine Algae—A Base for Inexpensive and Efficient Fertilizers
Source: Molecules. 2024 Mar 18;29(6):1348. doi: 10.3390/molecules29061348 (PMC10975981; doi:10.3390/molecules29061348)
Supplement: Supplementary file 1 [file molecules-29-01348-s001.zip › molecules-2898834-supplementary.pdf]

Removal of Arsenic and Its Inorganic Forms from Marine Algae—A Base for Inexpensive and Efficient Fertilizers

Table S1 Comparison of studied material with literature data regarding specific surface area and As(III)/As(V) adsorption performance

Material symbol	S_{BET} [$m^2\ g^{-1}$]	As(III) adsorption parameters	As(V) adsorption parameters	Literature
Hydroxyl-enriched CeO_2	-	$pH_0 = 3$, $t_{eq} = 40$ min, $A_{max} = 65.41$ mg g^{-1}	$pH_0 = 2$, $t_{eq} = 50$ min, $A_{max} = 71.91$ mg g^{-1}	[1]
Nano-CeO_2 aggregates	111	$pH_0 = 3$, $t_{eq} = 60$ min	-	[2]
Hydrous CeO_2 NPs	198	$pH_0 = 7$, $t_{eq} = 120$ min, $A_{max} = 170$ mg g^{-1}	$pH_0 = 7$, $t_{eq} = 120$ min, $A_{max} = 107$ mg g^{-1}	[3]
Engineered CeO_2 NPs	17	$pH_0 = 3.6$, $t_{eq} = 4320$ min, $A_{max} = 33.8$ mg g^{-1}	$pH_0 = 3.6$, $t_{eq} = 4320$ min, $A_{max} = 8.9$ mg g^{-1}	[4]
UiO-66-SH-A	30	$pH_0 = 9$, $t_{eq} = 30$ min, $A_{max} = 90.7$ mg g^{-1}	$pH_0 = 11$, $t_{eq} = 30$ min, $A_{max} = 98.8$ mg g^{-1}	[5]
ZCNs	-	$pH_0 = 6$, $t_{eq} = 25$ min, $A_{max} = 28.61$ mg g^{-1}	$pH_0 = 6$, $t_{eq} = 20$ min, $A_{max} = 106.57$ mg g^{-1}	[6]
CeO_2	187	$pH_0 = 4$, $t_{eq} = 600$ min, $A_{max} = 96.1$ mg g^{-1}	$pH_0 = 4$, $t_{eq} = 600$ min, $A_{max} = 68.5$ mg g^{-1}	This work

[1] Hu W., Yang L.,* Shao P. et al. Proton Self-Enhanced Hydroxyl-Enriched Cerium Oxide for Effective Arsenic Extraction from Strongly Acidic Wastewater, *Environ. Sci. Technol.*, ***2022***, *56*, 10412−10422, doi: 10.1021/acs.est.2c02675.

[2] Pang J.-H., Liu Y., Li J. et al. Solvothermal synthesis of nano-CeO_2 aggregates and its application as a high-efficient arsenic adsorbent, *Rare Met.*, ***2019***, *38*, 73-80, doi: 10.1007/s12598-018-1072-4.

[3] Li R., Li Q., Gao S. et al. Exceptional arsenic adsorption performance of hydrous cerium oxide nanoparticles: Part A. Adsorption capacity and mechanism, *Chem. Eng. J.*, ***2012***, *185-186*, 127-135, doi: 10.1016/j.cej.2012.01.061.

[4] Bi X., Zeng C. and Westerhoff P. Adsorption of Arsenic Ions Transforms Surface Reactivity of Engineered Cerium Oxide Nanoparticles, *Environ. Sci. Technol.*, ***2020***, *54*, 9437−9444, doi: 10.1021/acs.est.0c02781.

[5] Shao P., Ding L., Luo J. et al. Lattice-Defect-Enhanced Adsorption of Arsenic on Zirconia Nanospheres: A Combined Experimental and Theoretical Study, *ACS Appl. Mater. Interfaces*, ***2019***, *11*, 29736−29745, doi: 10.1021/acsami.9b06041.

[6] Luo J., Luo X., Hu C. et al. Zirconia (ZrO_2) Embedded in Carbon Nanowires via Electrospinning for Efficient Arsenic Removal from Water Combined with DFT Studies, *ACS Appl. Mater. Interfaces*, ***2016***, *8*, 18912−18921, doi: 10.1021/acsami.6b06046.

Table S2 Elemental composition of studied algae and BCR-279 before water extraction and elemental content extracted by water from these samples (* - mean value from 4 repeats, # - standard deviation from 4 repeats, LOD [mg kg^{-1}]: Cd (0.02), Pb (2.0), Se (4.0))

**Element**	**Total content [mg kg^{-1}]**				**Content extracted by water [mg kg^{-1}]**			
	**Sample 1**	**Sample 2**	**Sample 3**	**BCR 279**	**Sample 1**	**Sample 2**	**Sample 3**	**BCR 279**
Al	307*±17#	263*±8#	176*±23#	1465*±66#	6.86*±0.52#	4.61*±0,35#	4.05*±0.30#	1.88*±0.14#
As	34.3*±0.4#	55.5*±0.2#	58.9*±0.8#	3.10*±0.12#	21.6*±1.6#	35.2*±2.6#	40.5*±3.1#	0.75*±0.03#
B	109*±1#	118*±1#	115*±1#	60.1*±0.9#	87.4*±6.5#	21.2*±1.6#	85.6*±6.4#	44.0*±3.3#
Ca	9873*±102#	9795*±151#	2714*±82#	26,626*±144#	1289*±96#	1654*±124#	1350*±101#	1704*±127#
Cd	0.18*±0,01#	0.52*±0.01#	0.46*±0.03#	<LOD	<LOD	0.17*±0.02#	0.06*±0.01#	<LOD
Cr	23.0*±1.3#	1.88*±0.09#	1.70*±0.3#	6.97*±0.12#	0.28*±0.02#	0.15*±0.01#	0.17*±0.01#	0.09*±0.01#
Cu	1.18*±0.02#	1.41*±0.02#	1.13*±0.03#	11.8*±0.1#	0.27*±0.02#	0.27*±0.02#	0.21*±0.01#	3.56*±0.03#
Fe	565*±6#	517*±1#	277*±17#	2086*±26#	22.0*±1.5#	23.3*±1.4#	12.6*±1.0#	15.6*±1.1#
K	20,958*±324#	31,509*±315#	34,792*±107#	11,234*±128#	13,285*±723#	24,257*±996#	24,811*±820#	8926*±569#
Mg	7493*±84#	6556*±84#	6806*±22#	12,957*±66#	2363*±108#	3128*±96#	2670*±82#	5412*±115#
Mn	23.6*±0.2#	66.6*±0.3#	49.6*±1.4#	1860*±93#	6.45*±0.45#	18.9*±1.4#	15.0*±1.2#	239*±15#
Na	22,916*±324#	23,764*±340#	22,110*±182#	16,077*±91#	15,455*±243#	18,802*±305#	16,748*±358#	13,385*±385#
Ni	3.17*±0.05#	3.62*±0.06#	2.99*±0.19#	12.4*±0.3#	0.47*±0.03#	0.80*±0.06#	0.76*±0.05#	3.57*±0.23#
P	370*±6#	687*±1#	986*±7#	1825*±14#	220*±14#	428*±28#	639*±35#	974*±61#
Pb	<LOD	<LOD	<LOD	9.53*±0.09#	<LOD	<LOD	<LOD	<LOD
S	21,265*±258#	13,580*±197#	19,960*±222#	29883*±78#	6192*±364#	6228*±305#	7350*±426#	15,155*±912#
Se	<LOD	<LOD	<LOD	<LOD	<LOD	<LOD	<LOD	<LOD
Zn	22.8*±0.3#	20.6*±0.1#	27.6*±0.9#	41.5*±0.5#	10.7*±0.5#	4.79*±0.3#	10.6*±0.6#	3.35*±0.18#
